# The cost of acute respiratory infections in Northern India: a multi-site study

**DOI:** 10.1186/s12889-015-1685-6

**Published:** 2015-04-07

**Authors:** Samuel K Peasah, Debjani Ram Purakayastha, Parvaiz A Koul, Fatima S Dawood, Siddhartha Saha, Ritvik Amarchand, Shobha Broor, Vaibhab Rastogi, Romana Assad, Kaisar Ahmed Kaul, Marc-Alain Widdowson, Renu B Lal, Anand Krishnan

**Affiliations:** College of Pharmacy, Mercer University, 3001 Mercer University Drive, Atlanta, GA 30341-4155 USA; Centers for Disease Control and Prevention, Atlanta, USA; Centre for Community Medicine, All India Institute of Medical Sciences, New Delhi, 110029 India; Department of Internal and Pulmonary Medicine, Sheri Kashmir Institute of Medical Sciences, Soura, Srinagar, 190011 J&K India; Influenza Division, Centre for disease control and Prevention, US Embassy, Shantipath, Chanakyapuri, New Delhi, 110021 India; The INCLEN Trust, 2nd Floor, F-1/5, Okhla Industrial Area, Phase-I, New Delhi, 110020 India; Sheri Kashmir Institute of Medical Sciences, Soura, Srinagar, 190011 J&K India; GB Pant Hospital, Srinagar, India

**Keywords:** Acute respiratory infections, Costs, Outpatient, Inpatient, Direct, Indirect, Public, Private

## Abstract

**Background:**

Despite the high mortality and morbidity resulting from acute respiratory infections (ARI) globally, there are few data from low-income countries on costs of ARI to inform public health policy decisions We conducted a prospective survey to assess costs of ARI episodes in selected primary, secondary, and tertiary healthcare facilities in north India where no respiratory pathogen vaccine is routinely recommended.

**Methods:**

Face-to-face interviews were conducted among a purposive sample of patients with ARI from healthcare facilities. Data were collected on out-of-pocket costs of hospitalization, medical consultations, medications, diagnostics, transportation, lodging, and missed work days. Telephone surveys were conducted two weeks after medical encounters to ask about subsequent missed work and costs incurred. Costs of prescriptions and diagnostics in public facilities were supplemented with WHO-CHOICE estimates of hospital bed costs. Missed work days were assigned cost based on the national annual per capita income (US$1,104). Non-medically attended ARI cases were identified from an ongoing community-based ARI surveillance project in Faridabad.

**Results:**

During September 2012-March 2013, 1766 patients with ARI were enrolled, including 451 hospitalized patients, 1056 outpatients, and 259 non-medically attended patients. The total direct cost of an ARI episode requiring outpatient care was US$4- $6 for public and $3-$10 for private institutions based on age groups. The total direct cost of an ARI episode requiring hospitalized care was $54-$120 in public and $135-$355 in private institutions. The cost of ARI among those hospitalized was highest among persons aged > = 65 years and lowest among children aged < 5 years. Indirect costs due to missed work days were 16-25% of total costs. The direct out-of-pocket cost of hospitalized ARI was 34% of annual per capita income.

**Conclusions:**

The cost of hospitalized ARI episodes in India is high relative to median per capita income. Data from this study can inform evaluations of the cost effectiveness of proven ARI prevention strategies such as vaccination.

**Electronic supplementary material:**

The online version of this article (doi:10.1186/s12889-015-1685-6) contains supplementary material, which is available to authorized users.

## Background

Acute respiratory infections (ARI) are major public health threats, especially among children aged less than 5 years who are at risk for severe illness. In 2010, an estimated 120 million cases of pneumonia occurred globally and 11.9 million cases of acute lower respiratory infections (ALRI) were associated with hospitalization [1]. Additionally, 99% of estimated ALRI deaths occurred in developing countries [1]. In Asia, an estimated 1.3 million children aged less than 5 years die from ALRI annually [2]. Although most ARI studies in developing countries have focused on children, there are studies on adults, especially on influenza infections in the elderly [3]. ARIs can result from infection with bacteria such as *Streptococcus pneumoniae* and *Haemophilus influenzae* or viruses such as respiratory syncytial viruses and influenza viruses. Effective vaccines against *S pneumonia*, *H influenza*, and influenza viruses are now available and recommended in many high-income countries but rarely recommended in low-income countries [4]. Competing public health priorities, economic consideration, lack of public funding for vaccination, have been cited as some of the contributing factors for low adoption of influenza vaccination in developing countries [5].

In addition to causing substantial morbidity and mortality, ARI also result in economic losses through increased use of healthcare resources and loss in productivity. Costs-of-illness studies provide critical information on the economic impact of diseases, and inputs for evaluations of cost-effectiveness of interventions. Combined with burden of disease estimates, cost studies provide data on disease impact to guide public health policy decisions about evidence-based interventions for effectively addressing public health threats [3]. Although the cost of ARIs has been estimated in high-income countries[6-8], there are few data on costs in low or middle income countries where disease severity may be worse because of delayed health-care seeking and where out-of-pocket costs may have an even higher impact on already impoverished populations [4,9]. Most of such studies in Southeast Asia have been on pneumonia or influenza [10-13].

In India, the burden of ARI is high [14,15], but vaccines against common causes of ARI are not part of the national immunization schedule with the exception of measles vaccine.

We conducted a multi-site cost-of-illness study in three communities in India to document the economic impact of ARI-related hospitalizations and outpatient visits by age in public and private facilities, and patient costs for non-medically attended ARI since prior data suggest that many persons in India do not seek medical care for ARI [16,17].

## Methods

### Setting and sites

This study was conducted during September 2012 to March 2013 at selected primary, secondary and tertiary care facilities in the National Capital Region (New Delhi and Faridabad) and Srinagar in Jammu-Kashmir state of India. Twenty-four health facilities covering all three levels of medical care participated in the study, including 4 tertiary, 9 secondary and 11 primary care facilities. Six hospitals/clinics in Faridabad refused to participate including four private tertiary level hospitals, one private secondary level facility, and one public hospital.

Primary level facilities were defined as those providing outpatient and emergency care only and not overnight admissions. Secondary level facilities were defined as those that provide both outpatient and inpatient care but lack capacity to provide intensive care. Tertiary level facilities were those capable of providing intensive care.

The non-medically attended portion of the study was conducted only in the Faridabad site during the same period as the medically-attended study. Participants were from an ongoing epidemiological study of respiratory pathogens in four villages of Ballabgarh in Faridabad.

### Data collection

Data collectors visited each facility two to three times a week. Patients attending participating healthcare facilities on these days were eligible for enrollment if they provided consent and met the case definition for ARI. ARI was defined as per the European Center for Disease Prevention and Control case definition [18] as acute onset within seven days of at least one of cough, sore throat, shortness of breath or coryza, plus a clinician’s diagnosis of an infection. Hospitalized patients were eligible for enrollment if they were hospitalized at least overnight and ARI was the primary diagnosis for the admission.

For inpatients, administered questionnaires were through face-to-face interviews at admission and/or discharge, followed by a telephone interview two weeks after discharge. Inpatient face-to-face administered questionnaires collected data on out-of-pocket cost of admission, medical consultations, medications, diagnostic procedures, transportation, and lodging fees. The telephone interviews covered missed workdays of both patients and caregivers and any other information not available at discharge. For outpatients, administered questionnaires were through face-to-face interviews at the time of consultation, and followed up with a telephone call two weeks after the visit. Outpatient questionnaires collected data on consultation, self-purchased prescriptions, diagnostic procedures, and transportation. By definition, non-medically attended ARI cases are those where the patient fulfils the case definition of ARI and had not consulted any trained or untrained medical personnel for his/her disease throughout the episode. Questionnaires were administered to patients with non-medically attended ARI at the time of the household visit (if the episode is over) to collect data on duration of symptoms, cost of self-medications and any non-medical consultation (e.g. consultation with an herbalist), and time taken off work for the illness. Unlike the medically-attended cases, no follow-up telephone interviews were conducted since indirect cost was not considered.

### Valuation of costs

Direct cost was defined as medical cost (cost of admission, consultation, medications, or diagnostics such as radiologic and laboratory studies) and non-medical cost (transportation, and lodging fees). Direct cost components were self-reported by patients during in-person or telephone follow-up interviews. Indirect cost was defined as the monetary value of lost earnings of adult patients and caregivers of all age groups due to inability to perform regular duties because of illness. Estimation of indirect cost was based on the assumption that labor was replaced at a cost to maintain societal productivity.

### Analysis

For medically-attended patients, direct cost was calculated as the sum of the direct medical and non-medical cost of the current hospitalization and follow-up visits to health facilities for the same ARI episode. Costs paid by the patient were considered for medical encounters at private facilities and by the patient and government at public facilities. WHO-CHOICE estimates were used as a proxy for the cost to the government in public facilities. WHO-CHOICE estimates [19] are available for hospital bed-cost per day in public inpatient facilities or cost of outpatient visits in public outpatient facilities but exclude cost of diagnostic procedures and medications. We used the actual cost paid out-of-pocket by patients for diagnostic procedures and medications (confirmed by medical records) as a proxy for the cost of medications and diagnostic procedures in both private and public facilities.

Direct medical cost in public facilities is the sum of out-of-pocket cost paid by patients for diagnostic tests and medications and the WHO-CHOICE estimates. For inpatients, the cost to the government is the product of WHO-CHOICE estimates [19] per hospital day and the median days of hospitalization. For outpatients, WHO-CHOICE estimates for outpatient facilities were used in lieu of the consultation fee paid by the patient, because WHO-CHOICE estimates better estimate the resources used in these facilities than the token consultation fee paid by the patient.

In calculations involving WHO-CHOICE estimates, all public teaching hospitals were considered tertiary institutions and other public hospitals were considered secondary institutions. For outpatient estimates, WHO-CHOICE estimates for secondary level hospitals were used for all public facility visits. The 2008-based year estimates were adjusted to 2012 using the consumer price index (1.24) for India [20]. Non-medically attended ARI direct cost was calculated as the sum of cost of non-medical expenditures and self-purchased prescriptions.

Indirect cost was estimated as the product of missed work days and the median per capita income of US$1,104 per year [21]. Missed work days of the patient were estimated by adding two days to the reported length of hospital stay (similar to Molinari et al. [22]).

Costs were calculated by study site, age group, level of care, and institution type (public vs. private) to reduce skewedness of summary results. Medians and interquartile ranges for different subgroups are reported instead of means because data were not normally distributed. For example, the mean cost of admission in private facilities was $266 (SD $402) but the median was $166 (IQR $108-$276). The exchange rate used for conversion of Indian Rupees (INR) to US$ was 1INR = 0.016 US$ [23].

### Ethical review

The study protocol was reviewed and approved by the Institution Ethics Committee, All India Institute of Medical Sciences, Ansari Nagar, New Delhi (Protocol ID: 6296 IRB Registration number: IRB0000682 & FWA #00014191) and the Institutional Ethics Committee, Sher-i-Kashmir Institute of Medical Sciences, Srinagar (IRB00008643). The Institutional Review Board of the US Centers for Disease Control and Prevention relied on review of the Institution Ethics Committee of the All India Institute of Medical Sciences.

## Results

### Epidemiology

#### Non-medically-attended ARI

Overall, 259 participants with non-medically attended ARI were enrolled; 88 (34%) were children aged <5 years and 21 (8%) were adults aged > = 65 years (Table 1). Participant gender was generally evenly balanced in each age group except among participants aged 18-64 years, 85% of whom were women. The median duration of non-medically attended ARI from symptom onset to recovery varied from 7 to 9 days.

**Table 1:** **Characteristics and cost of acute respiratory infection among persons with non-medically attended acute respiratory illness, August 2012-March 2013, Faridabad district, India, N = 259**

	**<=5 years**	**6**- **17 years**	**18 to 64 years**	**> = 65 years**
	**n = 88**	**n = 70**	**n = 80**	**n = 21**
**Male**	48 (55%)	37 (53%)	15 (19%)	9 (43%)
**Unemployed/dependent**	-	-	2 (3%)	9 (43%)
**Pharmacy consultation** ^**1**^	25 (28%)	21 (30%)	17 (21%)	4 (19%)
**Purchased medication** ^**2**^	11 (13%)	6 (9%)	9 (11%)	3 (14%)
**Median days with symptoms (IQR)**	7 days	7 days	8 days	9 days
	(4-9 days)	(6-10 days)	(6-10 days)	(8-10 days)
**Median cost of medications (IQR)**	US$ 1.1	US$1.3	US$1.3	US$1.1
	($0.6-$1.9)	($1.3-$1.6)	($0.9-$2.4)	($0.4-$1.3)

^1^Pharmacy consultations are normally free because the fees are part of the cost of medications but reflect proportion who sort help from the pharmacy for over-the-counter medications.

^2^Number of people (%) who bought medications.

#### Medically-attended ARI (inpatient and outpatient)

Overall, 1,507 patients with medically-attended ARI were enrolled in the study, including 451 (30%) inpatients and 1,056 (70%) outpatients (Table 2). Persons aged >65 years accounted for the largest proportion of inpatients (259/451, 47%), whereas persons aged 18-64 years accounted for the largest proportion of outpatients (614/1056, 39%). Housewives and unskilled laborers were the most common occupation among both inpatients (25% and 19%, respectively) and outpatients (18% and 14%). The majority of inpatients (331/451, 73%) and outpatients (528/1056, 50%) received medical care at tertiary care facilities, and all participants from Srinagar were enrolled at tertiary care facilities. Overall, the median length of hospitalization among inpatients was 5 days, but median length of hospitalization was higher in Srinagar than the National Capital Region (9 days vs. 5 days). All of the inpatient cases were classified as severe lower respiratory infection by the admitting physicians. For outpatient cases majority were upper respiratory infections. For example, in the National capital region, 79% were regarded upper respiratory infections mainly febrile acute respiratory illness or influenza-like illnesses. Of the remaining 21% lower respiratory infections, 53% were considered severe illness (mainly pneumonia).

**Table 2:** **Baseline characteristics of inpatients and outpatients with acute respiratory infection, August 2012-March 2013, National Capital Region and Srinagar, India, N = 1507**

	**Total**		**National Capital Region** ^**4**^		**Srinagar**	
	**Inpatients**	**Outpatients**	**Inpatients**	**Outpatients**	**Inpatients**	**Outpatients**
	**n = 451(%)** ^**3**^	**n = 1056(%)** ^**3**^	**n = 215(%)** ^**3**^	**n = 739(%)** ^**3**^	**n = 236(%)** ^**3**^	**n = 317(%)** ^**3**^
**Age group**						
<=5 years	135 (30)	383 (36)	65 (30)	283 (38)	70 (30)	100 (31)
6-17 years	19 (4)	136 (13)	14 (7)	89 (12)	5 (2)	47 (15)
18-64 years	136 (30)	409 (39)	71 (33)	263 (36)	65 (28)	146 (46)
> = 65 years	161 (36)	131 (12)	65 (30)	107 (14)	96 (40)	24 (8)
**Male**	259 (57)	614 (58)	151 (70)	464 (63)	108 (46)	150 (47)
**Occupation**						
Housewife	66 (25)	159 (18)	13 (8)	78 (12)	53 (51)	81 (41)
Unskilled	49 (19)	118 (14)	28 (18)	87 (13)	21 (20)	31 (16)
Retired	34 (13)	41 (5)	19 (12)	39 (6)	15 (14)	2 (1)
Skilled	11 (4)	63 (7)	4 (3)	42 (6)	7 (7)	21 (11)
Unemployed	8 (3)	36 (4)	8 (5)	36 (5)	-	-
Others^1^	95 (36)	459 (48)	88 (54)	377 (58)	8 (8)	107 (31)
**Facility level**						
Primary	-	250 (24)	-	250 (34)	-	-
Secondary	120 (27)	278 (26)	120 (56)	278 (38)	-	-
Tertiary	331 (73)	528 (50)	95 (44)	211 (28)	236 (100)	317 (100)
**Facility type**						
Public	325 (72)	838 (79)	89 (41)	521 (71)	236 (100)	317 (100)
Private	126 (28)	218 (21)	126 (59)	218 (29)	-	-
**Length of stay** ^**2**^	5(IQR 4-10)	-	5(IQR 4-7)	-	9(IQR 6-12)	-

^1^Others include children and students ^2^Length of hospital stays Median (interquartile range) ^3^The percentage of respondents within a given variable: For example, 30% of all participants who answered the question on age were 5 years or younger ^4^National Capital Region Includes Delhi and Faridabad.

### Health resource utilization

#### Non-medically attended ARI

Of all 259 participants, 19-30% visited a pharmacy for possible over-the-counter medication consultation (depending on age group), and 9-14% bought medications after the consultation (Table 1).

#### Medically-attended outpatient ARI

Among outpatients, 22% (233/1056) had non-medical consultations prior to their outpatient visits, and 30% (317/1056) had medical consultations prior to the visit. Additionally, 23% (247/1056) of outpatients had follow-up visits after the current outpatient visit. Almost every outpatient visit resulted in costs for prescribed medications (97-100%), but very few outpatients underwent laboratory or radiological studies (1-15%). Although 13% (141/1056) of patients and 21% (225/1056) of their caregivers missed work due to participants’ illnesses, only 7% lost money due to missed work (Table 3).

**Table 3:** **Health resource utilization among outpatients with acute respiratory infection, August 2012-March 2013, National Capital Region and Srinagar, India, N = 1056**

	**<=5 years**		**6-17 years**		**18-64 years**		**> = 65 years**	
	**Public**	**Private**	**Public**	**Private**	**Public**	**Private**	**Public**	**Private**
	**n = 262**	**n = 118**	**n = 110**	**n = 26**	**n = 345**	**n = 64**	**n = 121**	**n = 10**
	**(%)** ^**1**^	**(%)** ^**1**^	**(%)** ^**1**^	**(%)** ^**1**^	**(%)** ^**1**^	**(%)** ^**1**^	**(%)** ^**1**^	**(%)** ^**1**^
**Prior Non-Medical Consultation**	43 (17)	39 (67)	24 (22)	4 (15)	83 (24)	12 (19)	25 (21)	3 (30)
**Prior Medical Consultation**	109 (41)	17 (14)	33 (30)	10 (38)	84 (24)	14 (22)	46 (38)	4 (40)
**Follow-up Medical Consultation**	76 (29)	31 (26)	39 (35)	6 (23)	54 (16)	5 (8)	35 (29)	1 (10)
**Medications Prescribed**	250 (98)	114 (97)	102 (98)	26 (100)	333 (97)	64 (100)	117 (98)	10 (100)
**Radiological Studies**	9 (3)	4 (3)	10 (9)	2 (8)	42 (12)	2 (3)	18 (15)	1 (10)
**Patient Missed Work**	-	-	-	-	125 (36)	6 (9)	9 (7)	1 (10)
**Caregiver Missed Work**	87 (34)	12 (10)	41 (38)	2 (8)	60 (17)	3 (5)	19 (16)	1 (10)
**Patient/Caregiver Lost Money**	5 (2)	5 (4)	1 (1%)	3 (12)	51 (15)	4 (6)	5 (4)	1 (10)

^1^The percentage of participants who answered ‘yes’ to the question (related to a given variable) with all participants who answered the question as the denominator.

#### Medically-attended inpatient ARI

Among hospitalized children aged < 5 years, the most common components of out-of-pocket cost at public facilities were post-discharge prescriptions (incurred by 89% of participants), laboratory studies (81%) and radiologic studies (84%) (Table 4). In private facilities, the most common components for children age < 5 years were laboratory studies (91%) and radiologic studies (74%). Compared to patients in the public sector, patients in the private sector were more likely to undergo laboratory investigation (83% vs. 94%, p < 0.01) but less likely to have a post-discharge prescription (87% vs. 66%, p < 0.01). Among inpatients in all age groups, admission to intensive care units was infrequent, except among adults aged > =65 years at private institutions (12/38, 32%).

**Table 4:** **Health resource utilization among inpatients with acute respiratory infection, August 2012-March 2013, National Capital Region and Srinagar, India, N = 451**

	**<=5 years**		**6 -17 years**		**18-64 years**		**> = 65 years**	
	**Public**	**Private**	**Public**	**Private**	**Public**	**Private**	**Public**	**Private**
	**n = 100**	**n = 35**	**n = 10**	**n = 9**	**n = 92**	**n = 45**	**n = 123**	**n = 38**
	**(%)** ^**1**^	**(%)** ^**1**^	**(%)** ^**1**^	**(%)** ^**1**^	**(%)** ^**1**^	**(%)** ^**1**^	**(%)** ^**1**^	**(%)** ^**1**^
**Prior Non-Medical Consultation**	50 (50)	4 (11)	3 (30)	2 (22)	29 (32)	8 (18)	41 (3)	1 (3)
**Prior Medical Consultation**	55 (55)	18 (51)	3 (33)	7 (78)	32 (36)	14 (31)	39 (33)	6 (16)
**ICU Admission**	4 (4)	2 (6)	2 (20)	-	3 (3)	6 (13)	1 (1)	12 (32)
**Post Discharge Prescription**	89 (89)	15 (43)	8 (89)	5 (56)	70 (76)	32 (71)	88 (86)	32 (84)
**Laboratory Studies**	81 (81)	32 (91)	6 (67)	9 (100)	63 (81)	41 (91)	88 (87)	37 (97)
**Radiological Studies**	84 (84)	26 (74)	8 (89)	7 (78)	50 (63)	40 (89)	64 (65)	37 (97)
**Caregiver Lodged**	6 (6)	2 (6)	-	1 (11)	2 (2)	1 (2)	3 (3)	4 (11)
**Patient Missed Work**	-	-	-	-	34 (37)	17 (38)	4 (3)	7 (18)
**Caregiver Missed Work**	77 (77)	9 (26)	7 (78)	1 (11)	58 (71)	21 (47)	77 (75)	24 (63)
**Patient/Caregiver Lost Money** ^**2**^	3 (3)	-	-	-	21 (26)	12 (27)	2 (2)	1 (3)

^1^The percentage of participants who answered ‘yes’ to the question (related to a given variable) with all participants who answered the question as the denominator.

^2^Reported that either patient or caregiver lost money because they missed work.

### Direct, indirect, and total cost of ARI

#### Cost of ARI in non-medically-attended settings

Since patients are not charged consultation fee at the pharmacy, the only reported cost was the cost of medications bought at the pharmacy. For those who bought medications, the median cost of medications was US$1.30.

#### Cost of ARI in outpatient settings

**Figure 1:**
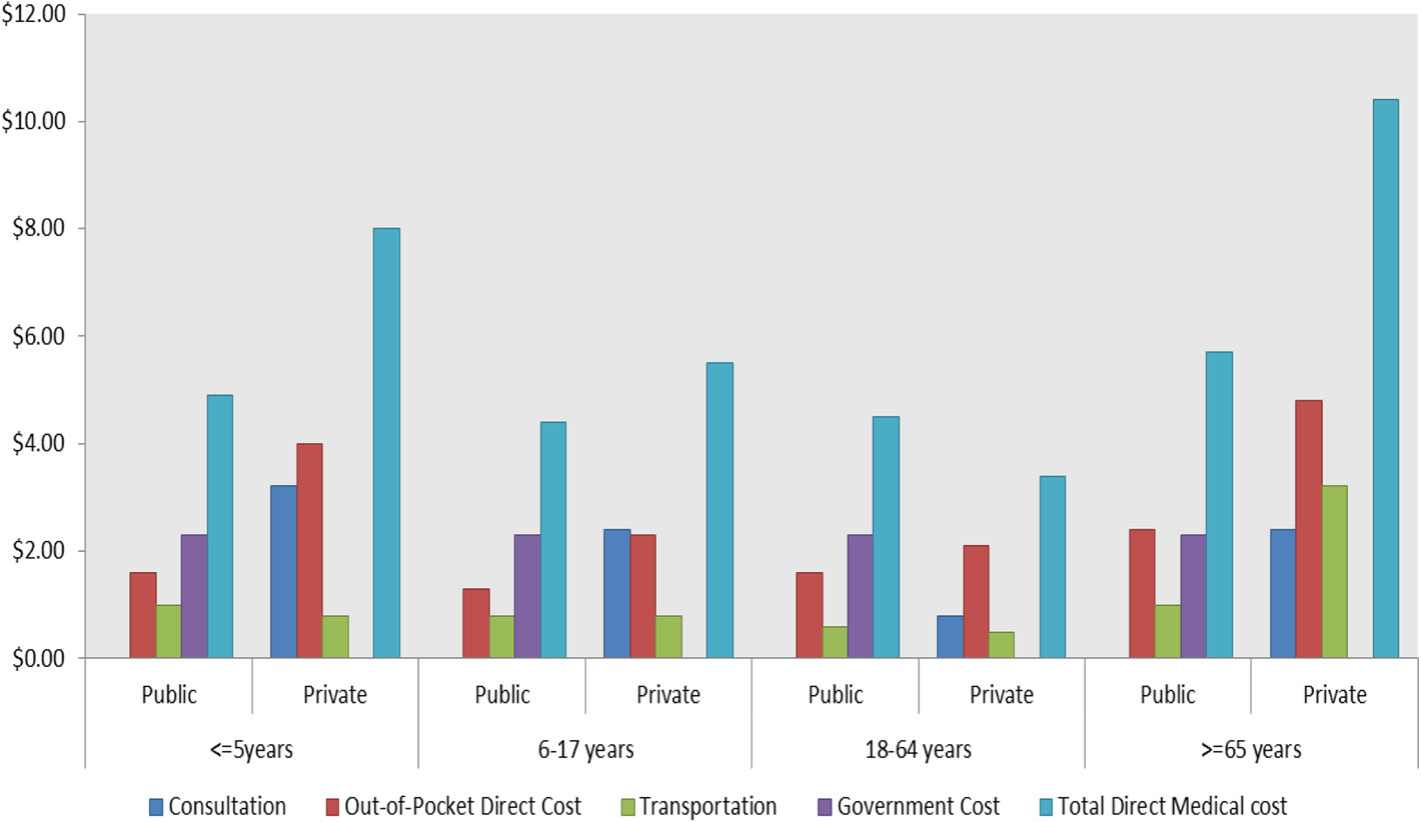
**Median Costs (US$) of outpatient-ARI in northern India by age and health sector.**

The total direct cost of ARI episodes requiring outpatient care was US$4-$6 for public and $3-$10 for private institutions based on age groups (Figure 1). Consultation costs in private facilities were similar to the WHO-CHOICE estimates in public facilities. However, out-of-pocket expenditures for direct medical cost in public facilities (~US$1-2) were lower compared to private institutions (US$2-5).

#### Costs of ARI in inpatient settings

Direct cost of ARI was twice as high in private (US$135-$355) as public (US$54-$120) institutions, 2.5 times higher in tertiary than secondary institutions, and increased with increasing age. Of all age groups, the median direct cost of ARI was highest in adults aged > =65 years in private facilities (US$355) and public facilities (US$120) (Additional file 1). Among children aged < 5 years, the median direct cost of ARI was US$135 in private and US$54 in public institutions. Direct cost was 2 to 20 times higher than indirect cost. As a proportion of median annual per capita income in India (US$1,104 in 2013), the total cost of ARI in inpatients ranged from 6% (IQR 5-9%) in secondary level public institutions to 34% (IQR 20-65%) in among adults aged > =65 years in private institutions. At public facilities, the estimated cost of a hospital-bed paid by the government accounted for 67% of the total direct cost of the hospitalization.

#### Indirect cost of ARI

Among outpatients, missed work days were most common among caregivers of children aged <5 years at public facilities (128/373, 34%) and least common among persons aged 17-64 years in private facilities (3/64, 5%) (Table 3). However, only 7% (75/1056) of all participants reported that they or their caregiver lost money due to missed work.

Among all inpatients, 14% (62/451) reported that they missed work due to their hospitalization and 61% (274/451) reported that a caregiver missed work. A larger proportion of children aged <5 years had a caregiver who missed work due to the illness at public facilities than private facilities (77% vs. 26%, p < 0.01) (Table 4). Among adults aged 18-64 years, 37% (51/137) reported missing work due to their illness, 58% (79/137) reported that a caregiver missed work due to their illness, and 24% (33/137) reported that either they or a caregiver lost money due to missed work.

Estimated indirect cost was higher in public facilities ($24-$34) than in private facilities ($21) because median length of hospitalization was longer in public compared to private facilities (7 vs. 4 days) resulting in more missed days of work. Indirect costs also accounted for a larger percentage of total costs in public compared to private facilities (25% vs. 16%).

## Discussion

Costs of acute respiratory infections studies in the Southeastern Asia region are scanty and most of the studies focus on pneumonia in children under 5 years or influenza. Our study sought to provide a comprehensive overview of the cost of ARI; in different age groups, at different levels of institutional care, in both private and public institutions, and uniquely, the cost of non-medically attended ARI cases. In addition, we compared the cost of ARI to the median per capita income for India to demonstrate the economic impact of ARI on patients and their families. Cost of ARI was substantial, particularly for ARI-associated hospitalizations which were associated with costs up to a third of median annual per capita income.

Costs incurred at private facilities were up to twice the costs incurred at public facilities; consistent with prior studies documenting higher costs at all levels of healthcare in the private sector compared to the public sector [24,25]. Although WHO-CHOICE estimates were added to the cost for public facilities to account for government costs; the disparities in inpatient cost between the two sectors remain substantial. Higher costs in the private sector may be driven by differences in treatment protocol, and contributors to higher costs in the private sector need to be identified.

The direct medical cost of ARI among inpatients varied substantially by age, similar to the findings of Zhou et al. [26] in China ($231 in children to $2,263 in older adults). The out-of-pocket inpatient cost was the highest among adults aged > = 65 years, meanwhile the majority of adults in this age group were either retired or indicated that they were housewives. Although children aged <5 years incurred the lowest costs per ARI episode in both public and private sectors, the burden of ARI pathogens (for example influenza-associated hospitalizations and outpatient visits) are high [4,27]. For example, although robust epidemiological data on ARI incidence in India are not available, about a fourth of total deaths in < 5 year olds are due to pneumonia [15]. Additionally, 90% of almost 150 million new episodes of pneumonia identified yearly occur in developing countries [28]. Therefore, overall cost of ARI among young children is likely to be high despite the lower relative cost we found in children. Immunization is listed as one of the potential interventions for preventing pneumonia but scanty cost data might be contributing to the lack of attention given to vaccination of at-risk groups in developing countries [29]. WHO’s Strategic Advisory Group of Experts on immunization (SAGE), recommends global influenza vaccination in targeted populations as an effective tool to mitigate the impact of influenza pandemics. Efforts are under way by the Global Vaccine Action Plan to promote and increase the capacity of developing countries for vaccination uptakes [30].

The indirect cost of ARI was substantially lower than the direct cost, and most participants reported not losing money from work absences. These findings are consistent with findings from other cost studies in Asia. In Hong Kong, Fitzner et al. found that indirect cost of influenza in Hong Kong [31] was low, because most participants visited the clinics during days off from work or after work and the US, estimated that indirect costs were approximately half of direct costs [17]. In contrast, Simmerman et al. [32] estimated that indirect and direct costs of influenza were roughly equal in Thailand, and studies from some European countries have documented indirect costs up to 10 times higher than direct costs [33]. Given that our sample consisted of a significant number of housewives and students who reported not losing money due to the illness, there is the need for further investigations into indirect cost of ARI in India since that can substantially affect the total cost of ARI.

Although the overall cost of hospitalized ARI at public facilities was largely driven by hospital-bed costs to the government, many patients also reported paying out-of-pocket for radiologic and diagnostic tests during hospitalization and for post-hospitalization prescription medications. Among patients with outpatient ARI and non-medically attended ARI (mainly upper respiratory infections), out-of-pocket costs were low with the median cost equivalent to 1-5% and 1% of the monthly per capita income in India, respectively. Cost of Inpatient ARI (mainly severe lower respiratory infections warranting admission), however, can be substantial (6-34% of annual per capita income in India).

Several limitations should be considered when interpreting our findings. We used a purposive sample (a subset sample with unique characteristics from a larger population) from ongoing surveillance studies (mainly from middle- to low-income clientele) and therefore, costs might not be representative of the population studied. Our study population also included a substantial number of housewives, retirees and students, adding to the complexity of estimating loss productivity. Out-of-pocket costs were self-reported and were not verified with medical records. Additionally, all data on non-medically attended ARI came from only the Faridabad site and therefore the findings do not represent the other two sites. Despite these limitations, we believe our study also has several strengths. This was a prospective study with a limited period of recall which minimized inaccurate recall of self-reported costs. Additionally, this study estimated costs in both the public and private sectors at all levels of care and among all age groups in the second highest populous nation globally.

## Conclusions

The cost of ARI episodes was substantial in our study communities, and should be considered when weighing public health priorities. Approaches to reduce the cost of treatment and strategies to reduce the morbidity load would be helpful. Proven strategies to reduce the burden including prevention strategies like influenza and pneumococcal vaccination, should be encouraged and pursued. Our data suggesting higher costs in older adults and children can be considered in age-group-specific prevention and treatment recommendations.

## Additional file

Additional file 1: Figure S1. Median Costs (US$) of Inpatient-ARI in Northern India by Age and Type of Facility. **Table S1.** Median costs (and IQR) in 2012 US$ of acute respiratory infection requiring outpatient care by age, August 2012-March 2013,National Capital Region and Srinagar, India, N=1056. **Table S2.** Median costs (and IQR) in 2012 US$ of acute respiratory infections (ARI) requiring inpatient care by type of facility, level of care, and age group, August 2012-March 2013, National Capital Region and Srinagar, India, N=451.

## Abbreviations

ARI: Acute respiratory infection
ALRI: Acute lower respiratory infection
WHO-CHOICE: World Health Organization’s- **CHO**osing **I**nterventions that are **C**ost-**E**ffective
IRBs: Institutional review boards
MAARI: Medically-attended ARI
