# Supplementary material for: The cost of acute respiratory infections in Northern India: a multi-site study
Source: BMC Public Health. 2015 Apr 7;15:330. doi: 10.1186/s12889-015-1685-6 (PMC4392863; doi:10.1186/s12889-015-1685-6)
Supplement: Additional file 1: Figure S1. — Median Costs (US$) of Inpatient-ARI in Northern India by Age and Type of Facility. Table S1. Median costs (and IQR) in 2012 US$ of acute respiratory infection requiring outpatient care by age, August 2012-March 2013,National Capital Region and Srinagar, India, N=1056. Table S2. Median costs (and IQR) in 2012 US$ of acute respiratory infections (ARI) requiring inpatient care by type of facility, level of care, and age group, August 2012-March 2013, National Capital Region and Srinagar, India, N=451. [file 12889_2015_1685_MOESM1_ESM.docx]

Additional file

Figure S1: Median Costs (US$) of Inpatient-ARI in Northern India by Age and Type of Facility

Table S2 Median costs (and IQR) in 2012 US$ of acute respiratory infection requiring outpatient care by age, August 2012-March 2013,National Capital Region and Srinagar, India, N=1056

|  | <=5 years | | 6-17 years | | 18-64 years | | >=65 years | |
| --- | --- | --- | --- | --- | --- | --- | --- | --- |
|  | Public  n=262 | Private  n=118 | Public  n=110 | Private  n=26 | Public  n=345 | Private  n=64 | Public  n=121 | Private  n=10 |
| Consultation | - | 3.2  (2.4-3.2) | - | 2.4  (0.8-3.2) | - | 0.8  (0.8-1.6) | - | 2.4  (0.8-2.4) |
| Out-of-pocket patient direct medical cost^1^ | 1.6  (0.6-3.2) | 4.0  (2.4-6.0) | 1.3  (0.8-4.0) | 2.3  (0.4-17.7) | 1.6  (0.6-4.0) | 2.1  (0.8-4.8) | 2.4  (0.9-6.0) | 4.8  (2.7-6.4) |
| Transportation cost | 1.0  (0.5-1.6) | 0.8  (0.5-1.6) | 0.8  (0.6-2.4) | 0.8  (0.3-1.1) | 0.6  (0.3-1.6) | 0.5  (0.2-0.8) | 1.0  (0.6-2.4) | 3.2  (3.2-3.2) |
| Estimated government cost^2^ | 2.3 | - | 2.3 | - | 2.3 | - | 2.3 | - |
| Total direct medical^3^ | 4.9 | 8.0 | 4.4 | 5.5 | 4.5 | 3.4 | 5.7 | 10.4 |

1. These are cost post visit and it includes prescriptions, diagnostics (X-ray/labs) bought after the visit
2. Replaces consultation fee in public facilities
3. Direct medical cost is the sum of consultation, out of pocket direct medical, and transportation cost.

Table S3 Median costs (and IQR) in 2012 US$ of acute respiratory infections (ARI) requiring inpatient care by type of facility, level of care, and age group, August 2012-March 2013, National Capital Region and Srinagar, India, N=451

|  | | Direct out-of-pocket cost^1^ | Estimated government cost^2^ | Total direct cost^3^ | Indirect cost to society^4^ | Total cost of ARI |
| --- | --- | --- | --- | --- | --- | --- |
| Level of Facility | | | | | | |
| Secondary level care | Public  (n=36) | 4  (2-12) | 38  (31-46) | 42  (33-58) | 24  (21-27) | 66  (54-85) |
|  | Private  (n=84) | 149  (114-232) | - | 149  (114-232) | 21  (17-24) | 170  (131-256) |
| Tertiary level care | Public  (n=289) | 31  (11-112) | 80  (49-119) | 111  (60-231) | 34  (24-48) | 145  (84-279) |
|  | Private  (n=42) | 393  (237-744) | - | 393  (237-744) | 21  (17-27) | 414  (254-771) |
| Age Group | | | | | | |
| 0-5 years | Public  (n=100) | 10  (4-33) | 44  (26-70) | 54  (30-103) | 24  (17-34) | 78  (47-137) |
|  | Private  (n=35) | 136  (103-185) | - | 135  (103-185) | 21  (17-24) | 156  (120-209) |
| 6-17 years | Public  (n=10) | 21  (6-52) | 44  (26-79) | 65  (32-131) | 24  (17-38) | 89  (49-169) |
|  | Private  (n=9) | 151  (116-167) |  | 151  (115-167) | 21  (21-24) | 172  (136-191) |
| 18-64 years | Public  (n=92) | 33  (19-152) | 83  (27-48) | 116  (46-200) | 39  (27-48) | 155  (73-248) |
|  | Private  (n=45) | 208  (139-340) | - | 208  (139-340) | 21  (17-24) | 229  (156-364) |
| >=65 years | Public  (n=123) | 41  (19-144) | 79  (53-105) | 120  (72-249) | 38  (27-48) | 158  (99-297) |
|  | Private  (n=37) | 355  (208-690) | - | 355  (208-690) | 21  (21-24) | 376  (229-714) |

1. Direct out-of-pocket cost includes admission, prescriptions, diagnostics (X-ray and Lab work) and transportation expenses
2. WHO CHOICE estimates (adjusted to 2013) multiplied by the length of stay. The average of secondary and tertiary level was used in the age group estimates
3. Total direct cost is the sum of direct out-of-pocket cost and estimated government cost-of –bed charges
4. Indirect cost is the per capita income per day multiplied by the sum of length of hospital stay plus 2 days
